# Gamma irradiation of ocular melanoma and lymphoma cells in the presence of gold nanoparticles: *in vitro* study

**DOI:** 10.1002/acm2.12336

**Published:** 2018-04-29

**Authors:** Mozhgan Rezaei Kanavi, Somayeh Asadi, Sahar Balagholi, Fatemeh Alikarami, Hassan Nosrati, Hamid Ahmadieh

**Affiliations:** ^1^ Ocular Tissue Engineering Research Center Shahid Beheshti University of Medical Sciences Tehran Iran; ^2^ Ophtalmic Research Center Shahid Beheshti University of Medical Sciences Tehran Iran; ^3^ Department of Hematology Faculty of Allied Medicine Tehran University of Medical Science Tehran Iran; ^4^ Department of Hematology Faculty of Allied Medicine Iran University of Medical Science Tehran Iran; ^5^ Radiation Oncology Research Center Cancer Institute Tehran University of Medical Sciences Tehran Iran

**Keywords:** Burkitt's lymphoma cells, choroidal melanoma, gold nanoparticles, *in vitro*

## Abstract

The aim of this work was to determine whether conjugation of cultivated choroidal melanoma and Burkitt's lymphoma cells with gold nanoparticles (GNPs) is beneficial for these series of ocular cancer patients. GNPs are radiosensitizers and can sensitize tumors to radiotherapy.This application has been examined in several tumor types, but not in choroidal melanoma. This study shows the results of *in vitro* study on the choroidal melanoma and also Burkitt's lymphoma cells in the presence of GNPs during continuous gamma irradiation. Cytotoxicity of GNPs were assessed for five different concentrations then cultured melanoma and Burkitt's lymphoma cells were irradiated with a Gamma source in the presence and absence of NPs. Incubation of melanoma cells with GNP concentrations below 100 *μ*g/ml, accompanied by gamma irradiation, increased cell death (*P* value = 0.016) . In the absence of irradiation, GNPs at these concentrations did not affect cultured melanoma cell metabolism. Reduced cell viability resulted from a significant increase in absorbed energy by the tumor. Moreover, GNP concentrations higher than 200 *μ*g/ml induced cytotoxicity in melanoma cells. Cytotoxicity assay in GNPs‐loaded Burkitt's lymphoma cells showed a slight decrease in cell viability at 50 *μ*g/ml and clear cytotoxicity at concentrations higher than 100 *μ*g/ml (*P* value = 0.035). Concentration and proper injection doses of GNPs in sensitive tissues such as the human eye are important variables yet to be determined.This is the first report of choroidal melanoma dosimetry performed in the presence of GNPs and provides valuable insights into future therapeutic approaches. Further *in vitro* study with more different sizes and concentrations is needed to determine the optimum size and concentration before any clinical research in this regard.

## 1 INTRODUCTION

Choroidal melanoma^1^ is the most common eye cancer which originating from melanin‐containing cells and has the highest rate of metastasis among intraocular tumors.^2^, ^3^, ^4^, ^5^‎ ‎The most common method of treatment for choroidal melanoma is radiation therapy.^6^‎ ‎Experimental and Monte Carlo (MC) studies have investigated the dosimetry of choroidal melanoma using different radiotherapy sources.^7^, ^8^, ^9^

In radiation therapy, X and gamma rays, and beta and alpha particles can be emitted from radiation device such as linear accelerators, sealed radioactive sources, or from radiolabeled substances. Plaque brachytherapy is the most widely applied technique for choroidal melanoma radiotherapy. Radioactive plaques are placed on the exterior of the tumor to deliver high‐dose radiation to the tumor while reducing radiation exposure in surrounding healthy tissues.^10^, ^11^, ^12^‎ ‎However, healthy and tumor tissues follow the same specific algorithm of radiation dose absorption, which is a regular obstacle in the treatment of eye tumors.

Gold nanoparticles (NPs) have strong photoelectric absorption owing to their high atomic number and electron density, making them potential radiosensitizer. Secondary electrons and Auger electrons caused by gamma or x‐ray irradiation can produce very high local ionization density.^13^ Increasing the irradiation dose absorbed by the tumor reduces treatment time. Given that the tumor and nearby healthy tissues follow the same energy absorbance pattern, reduce treatment time lower the irradiation dose absorbed by healthy tissues and results in reduced adverse effects. However, if the NPs penetrate to the surrounding normal cells, the absorbed dose by these healthy cells will increase and this method does not benefit the ocular cancer patient. Our previous work demonstrated the proper distribution of GNPs within the eye tumor and showed that GNPs were not observed in extratumoral areas.^14^

Previously, it has been shown that loading gold NPs into prostate, skin melanoma, and liver tumors, which are later exposed to irradiation, results in greater dose absorption within the tumor than the surrounding tissues.^15^, ^16^, ^17^, ^18^

In recent MC studies,^19^, ^20^‎ ‎the effects of GNPs on the brachytherapy for choroidal melanoma, have been investigated using iodine (^125^I) and palladium (^103^Pd), the most common irradiation sources for this type of intraocular tumor. These simulations were performed with the eye model phantom and the water phantom to determine the dose enhancement factor (DEF) in the presence and in the absence of GNPs using the mentioned brachytherapy sources. In addition, the significance of the eye model on the DEF calculation was evaluated by comparing the dosimetry calculations in the presence of GNPs in both eye model phantom and water phantom. These simulations were done using the MCNP5 MC code and reported the absorbed dose enhancement by choroidal melanoma tumors for the mentioned sources. In fact the use of such brachytherapy sources along with GNPs would yield a higher DEF in the tumor site of the eye melanoma.

We performed an *in vitro* study on human choroidal melanoma and Burkitt's lymphoma cells and the comparison was made between them. To the best of our knowledge, this is the first time that choroidal melanoma dosimetry has been performed with experimental methods in the presence of GNPs.

*In vitro* analysis of the toxicity, radiosensitivity and the death of cells was performed by multiple MTT and cell death assay.^21^, ^22^‎ ‎In the *in vitro* phase, cultured melanoma cells were incubated with GNPs and were irradiated with Gamma source to observe the induced effects of the radioactive source through measuring cell death. Cytotoxicity and optimum GNP concentrations were examined using five different GNP concentrations. The same analysis was performed on Burkitt's lymphoma cells using MTT assay. Furthermore, the response to radiation of GNP‐loaded lymphoma and choroidal melanoma cells were studied through with cell death assay and compared.

## 2 METHODS

### 2.A Nanoparticle synthesis

Gold NPs were synthesized following the FERN method^23^ by employing chloroauric acid (HAuCl4‐gold halides) (Merck, Darmstadt, Germany), which was then reduced by sodium citrate. HAuCl4 compound was dissolved in an adequate volume of water, to obtain the desired 0.2145 m solution. The noted substance was heated, and a 1.26544 m sodium citrate was added.

The size of the resultant NPs can be controlled by the amount of added citrate. In this reaction, citrate is used to convert Au (III) to Au (I), and create the NPs. By shifting some factors such as temperature, and the ratio of the administered compounds, it is possible to control both the size, and the distribution of the NPs.^24^, ^25^, ^26^

**Figure 1:**
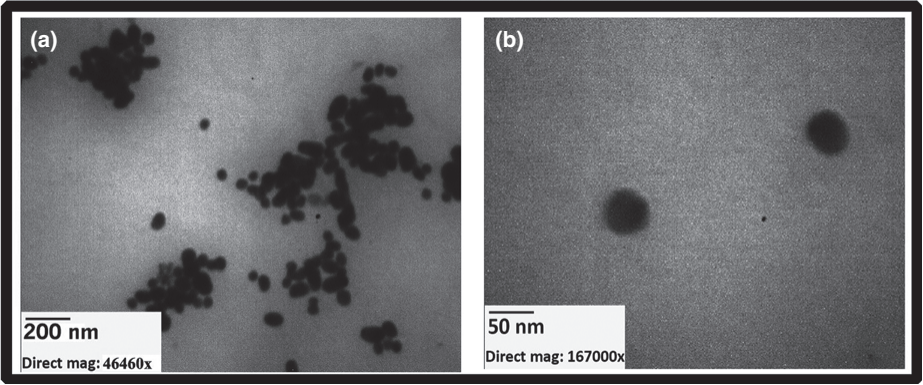
Transmission electron microscopy images of synthesized gold nanoparticles with average size of 50 nm. (a) Bar indicated 200 nm and (b) bar indicated 50 nm.

Transmission electron microscopy (TEM) was used to evaluate the morphology and size of synthesized gold NPs using a Zeiss‐EM10C‐80 KV electron microscope (Oberkochen, Germany) (Fig. 1). The size of gold NPs was determined by measuring the diameter of whole particles on the TEM image. The average size of these NPs was in the range of 50 nm and most were spherical in shape.

**Figure 2:**
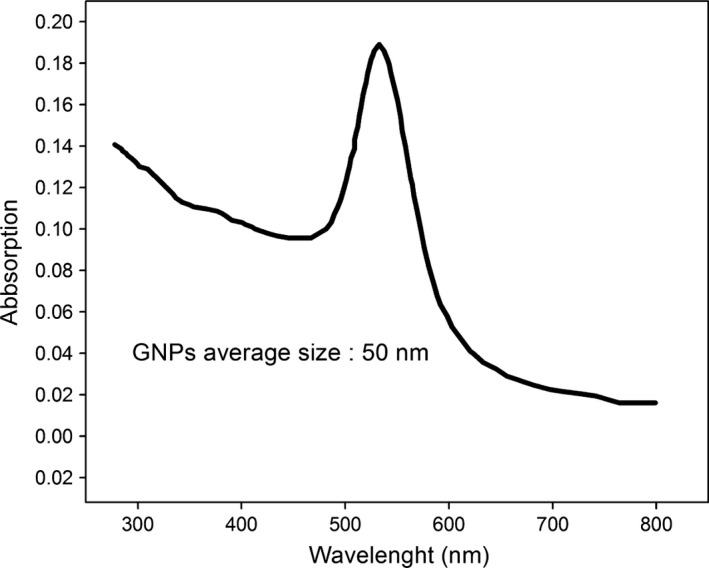
The UV‐visible absorption spectra of 50 nm GNPs; the maximum absorbance intensity of nanoparticles was in wavelength of around 532 nm.

Absorption spectra and absorbance measurements were obtained with a PerkinElmer UV‐visible spectrophotometer model Lambda 25 (Waltham, MA, USA) (Fig. 2). The absorbance measurement was made over the wavelength range of 200–700 nm using 3 ml SUPRASIL UV Quartz cells. A digital pH meter, model 632, Metrohm (Herisau, Switzerland), with a combined glass electrode was used for pH measurements.

### 2.B Cell culture and cytotoxicity assay

Melanoma cells were extracted from a middle‐aged human female who underwent enucleation for a very advanced malignant choroidal melanoma. Patient was a 62‐year‐old female with a clinical diagnosis of large choroidal tumor with no history of brachytherapy and suspicious to extras clearly extension of the tumor.

Histopathologic examinations disclosed an epithelioid type choroidal melanoma with extrascleral extension. Immediately after enucleation, the tumor tissue was obtained and digested with 1.25% trypsin and cultivated in Dulbecco's modified Eagle's medium F12 in a T75 flask with 20% fetal bovine serum (FBS). Cells were incubated at 37°C in a 5% CO_2_ atmosphere.

**Figure 3:**
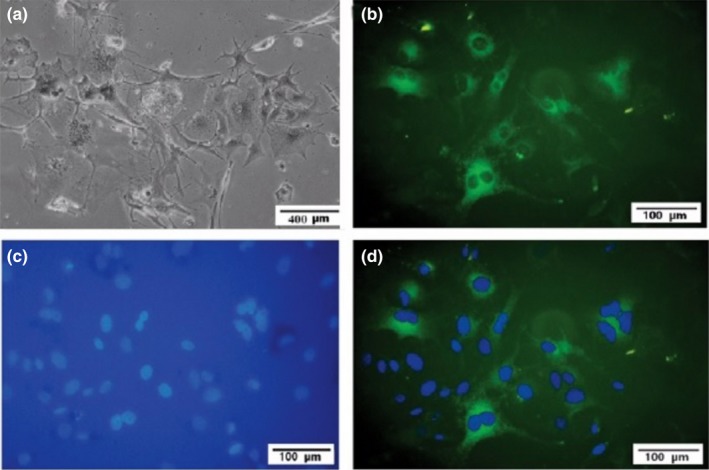
Representative cultivated human uveal melanoma cells: (a) Cultivated melanoma cells. (b) Stained positively for the fluorescein isothiocyanate (FITC)‐conjugated Melan‐A antibody (green). (c) Note DAPI‐stained melanoma cells nuclei in blue color and (d) merged image (FITC‐labeled Melan‐A and DAPI).

Immunocytochemistry for Melan‐A monoclonal antibody, as a melanoma marker, was performed to identify melanoma cells. Melanoma cells were incubated with mouse anti‐human Melan‐A antibody (M7196; Dako, Glostrup, Denmark) for 24 h at 4°C. Fluorescein isothiocyanate (FITC)‐conjugated antibody (goat‐anti‐mouse IgG; Santa Cruz, Carlsbad, CA, USA) was used for 45 min to detect the immunoreactivity of cultivated melanoma cells to the Melan‐A. All experiments were performed in duplicate. Cells were incubated with 4, 6‐diamidino‐2‐phenyindole dihydrochloride (DAPI; 1.5 mg/ml; Santa Cruz) for 10 min, and immunoreactive cells were observed by using an inverted microscope (Olympus IX71; Olympus Corporation, Tokyo, Japan) equipped with a 460 nm filter for DAPI and a 520 nm filter for FITC‐conjugated antibodies (Fig. 3).

**Figure 4:**
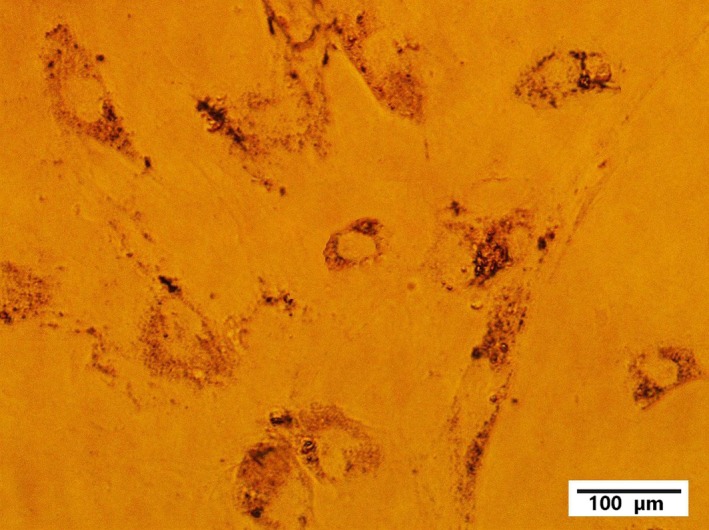
Light microscopy image of melanoma cells 24 h after treatment with GNPs.

At passage 5, melanoma cells (in the culture medium) were dispensed into 24‐well tissue culture plates (1 ml containing 27 × 10^5^ per well). GNPs (1 ml) were added to each well with concentrations of 50–600 *μ*g/ml. One well did not receive any GNPs, and was the control. Different concentrations (50, 100, 200, 400, or 600 *μ*g/ml) of GNPs were added to the other five wells. Figure 4 shows a confocal microscopy image of GNPs‐loaded melanoma cells taken 24 h after the addition of GNPs and showing that the GNPs were clustered in the cytoplasm.

Ramos cells (Burkitt's lymphoma‐derived cell line) were cultured in Roswell Park Memorial Institute 1640 medium supplemented with 10% FBS, 100 U/ml penicillin, 100 *μ*g/ml streptomycin, and 0.05% l‐glutamine serum in a T75 flask. Cells were dispensed into 24‐well tissue culture plates (1 ml containing 2 × 10^5^ cell per well) and incubated at 37°C with 5% CO_2_. GNPs were added to the cells as detailed above.

Cytotoxicity induced by different concentrations of GNPs was measured using a multiple MTT assay test (a quantitative colorimetric method to determine cytotoxicity) in a multiwell plate. Cell viability, expressed as percentage relative to the untreated control (100% cell viability), was measured in triplicate.

### 2.C Irradiation

Melanoma tumor or Burkitt's lymphoma cells were cultured in four 24‐well tissue culture plates and 50, 100, 200, 400, or 600 *μ*g/ml GNPs were added,;this experiment was performed in triplicate. The first wells in all four plates were the control samples and irradiated in the absence of NPs.

The plates were exposed to 30 Gy radiation using a Cobalt 60 machine, producing two gamma rays with energies of 1.17 and 1.33 MeV (Theratron 780; Phoenix Company, Ottawa, Canada). Cells were placed on Plexiglas (5 mm thick) in the center of the 30 × 30 cm^2^ field 80 cm from the irradiator so that the beam was homogenized.

The cells were then stored for 24, 72, and 168 h, before cell death was assessed. For apoptosis detection, a Cell Death Plus ELISA kit was used according to the manufacturer's instructions (Roche Molecular Biochemicals, Indianapolis, IN, USA). The experiment was repeated two more times with the same conditions except that cells were irradiated with doses of 10 and 20 Gy.

## 3 RESULTS

**Figure 5:**
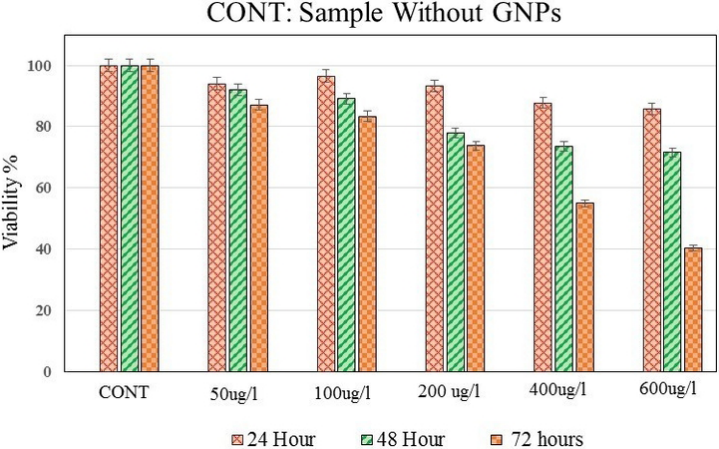
Viability comparison of melanoma cells exposed to different concentrations of GNPs as an indicator of GNPs toxicity in human eye melanoma cells. Cell viability was measured by the MTT assay test. Viability was calculated as the percentage of the viable cells compared to the untreated controls (CONT). The error bars represent the standard deviation of the mean of three replicates.

Melanoma cells were exposed to 50, 100, 200, 400, or 600 *μ*g/ml GNPs and cell viability was measured and compared to evaluate the cytotoxicity of the gold NPs (*P* value = 0.035) (Fig. 5). The highest concentration of GNPs that could be incubated with the cells, without causing visible damage to the cells, was determined in this figure.

**Figure 6:**
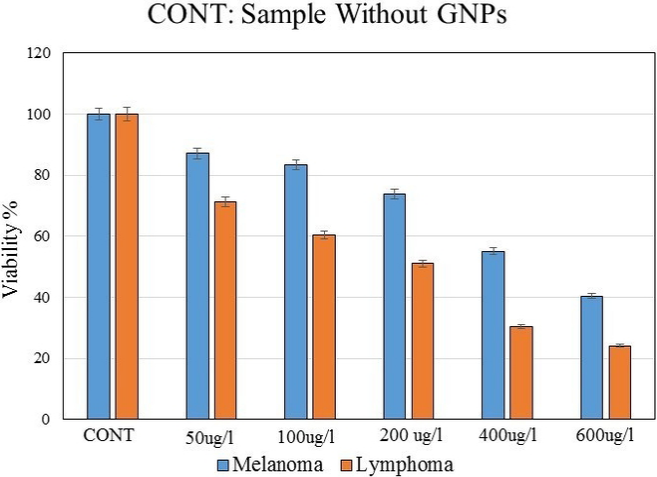
Comparison of the cytotoxicity induced by different concentrations of GNPs in the melanoma and Burkitt's lymphoma cells on third day after incubation. The error bars represent the standard deviation of the mean of three replicates.

The cytotoxicity induced in melanoma cells by different concentrations of GNPs was compared with that observed in Burkitt's lymphoma cells 3 days after the introduction of GNPs (Fig. 6).

We compared five different GNP concentrations in melanoma and Burkitt's lymphoma cells. Some concentrations resulted in a 50% or greater level of cell death in both kinds of cancer cells. While such concentrations cannot be used in a clinical radiation setting, the present work is a nonclinical primary study to investigate the behavior of GNP‐smeared cells that are exposed to radiation. Therefore, such concentrations could provide useful information about the use of GNPs in treating and targeting tumors and were used in the radiation phase of this study. Using this approach, we could identify the extent to which the death of eye melanoma cells in the absence of radiation could affect, or decrease, the viability of the cells when exposed to gamma radiation. Therefore, these results provide an assessment of cell death and allow us to investigate the energy absorbed by the tumor in proportion to the concentration of GNPs. These results physically report the increase of the energy released inside the cell for the higher concentrations of GNPs used. While the use of high GNP concentrations are not clinically relevant, they have been used in this study to provide invaluable information about how irradiation and GNPs interact to improve tumor targeting efficiency in cancer cells.

**Figure 7:**
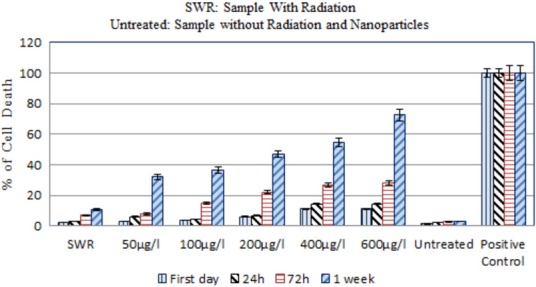
Cell death analysis of 30 Gy irradiated GNPs‐loaded melanoma cells with the cobalt source. Cell death assay shows increase in apoptosis of melanoma cells with increase in concentration of GNPs. This assay shows the most effects of GNPs on cell death in ‎‎600 *μ*g/ml concentration. The error bar represent the standard deviation of the mean of three replicates.

Melanoma cells that had been loaded with different concentrations of GNPs were irradiated with 30 Gy and the radiation‐induced apoptosis was quantified (Fig. 7) (*P* value = 0.016). We also applied two radiation doses of 10 and 20 Gy and the cell death was assayed 24 h after irradiation. The decreases of about 9% and 14% in cell viability were observed for melanoma cells in radiation doses of 10 and 20 Gy, respectively. Also a <20% decrease in cell viability was observed for lymphoma cells in these radiation doses. In comparison with the control sample, no significant differences in apoptosis were observed.

**Figure 8:**
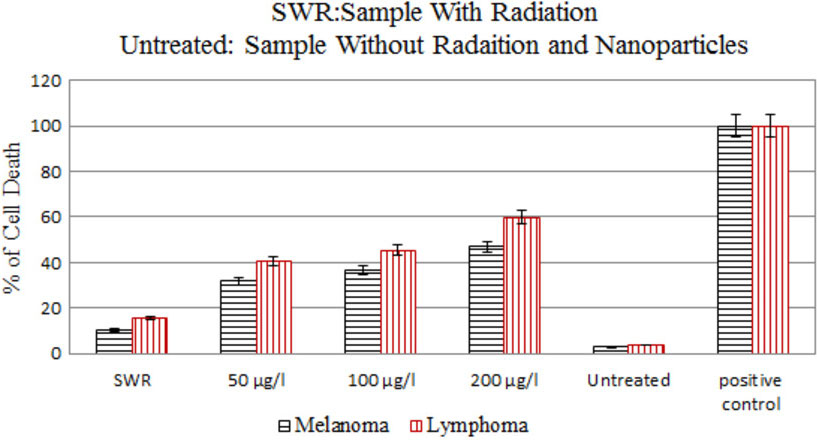
Comparison of the apoptosis for melanoma and Burkitt's lymphoma cells on the seventh day after irradiation for 50, 100, and 200 *μ*g/ml concentrations of GNPs. The error bars represent the standard deviation of the mean of three replicates.

Apoptotic response of GNPs‐loaded (50, 100, and 200 *μ*g/ml) melanoma and Burkitt's lymphoma cells to irradiation was compared on the seventh day after 30 Gy irradiation (Fig. 8).

## 4 DISCUSSION

Regarding the results of our experimental study (Fig. 7), the amount of increase observed in the number of dead melanoma cells irradiated in the presence of GNPs, compared to the amount of irradiated dead cells in the absence of GNPs can demonstrate the changes in absorbed energy.

It should be noted that factors including the number of cultured cells in a laboratory environment, the precise concentration of GNPs, equipment inaccuracy, human error, and other confounding factors, contribute to the inconsistencies that may be observed between experimental (*in vitro*) data and clinical calculation.

The main purpose of radiation therapy is to enhance the radiation dose to the tumor while sparing the healthy tissue. Given the radiation dose needed to halt the growth of choroidal melanoma in a specific time interval, administration of GNPs would increase tumor dose absorption. Increasing the radiation dose absorbed by the tumor leads to an increase the number of dead cancer cells and a decrease in treatment time. Furthermore, the radiation dose absorbed by normal tissues will decrease as a result of decreased treatment time.

GNPs cytotoxicity assays (Fig. 5) showed that GNP concentrations of 50 and 100 *μ*g/ml have no obvious cytotoxicity in melanoma cells, and the highest cytotoxicity was observed at a concentration of 600 *μ*g/ml.

Comparison of the results of melanoma and Burkitt's lymphoma cell cytotoxicity tests (Fig. 6) revealed that different concentrations of GNPs do not have the same effect on cell viability in both cancer cell types. Concentration of 200 *μ*g/ml showed toxicity effects in both melanoma and lymphoma cells however, in this concentration, a slight decrease of about 25% in cell viability was observed in melanoma cells, whereas in Burkitt's lymphoma cells, the same GNP concentration resulted in a >50% decrease in viability. A significant decrease in cell viability, of about 45% and 60%, was observed when melanoma cells were incubated with 400 and 600 *μ*g/ml GNPs, respectively. In Burkitt's lymphoma cells treated with 400 and 600 *μ*g/ml GNPs, the decrease in cell viability was more than 70% at both concentrations. Therefore, cellular cytotoxicity responses are cell type specific. Furthermore, our results show that GNPs in concentrations lower than 100 *μ*g/ml can be reliably incubated with choroidal melanoma cells without causing visible cell damage.

We observed about 8% and 12% increase in cell death in melanoma and Burkitt's lymphoma cells, respectively, 7 days after irradiation in the absence of GNPs, compared with untreated cells (Figs 7 and 8).‎ ‎Compared to controls (untreated cells), in the presence of 50, 100, and 200 *μ*g/ml GNPs cell death increased by about 29%, 34%, and 44%, respectively for melanoma cells and 36%, 41%, and 56%, respectively for lymphoma cells (Fig. 8). While concentrations of 50 and 100 *μ*g/ml GNPs alone have no obvious cytotoxic effects in melanoma cells, cell metabolism was significantly reduced when the cells were exposed to irradiation.

Here, we performed cytotoxicity assays comparing five concentrations of GNPs in choroidal melanoma cells. Additionally, we compared the combined effects of these GNP concentrations and three irradiation doses on choroidal melanoma and Burkitt's lymphoma cells. Taken together these results shows that radiation therapy combined with GNPs would need to be specifically tailored for each cancer type.

Considering the sensitivity of the human eye, determination of more precise optimum concentrations for GNPs and irradiation doses for choroidal melanoma treatment will require further studies including more GNP concentrations and a wider range of irradiation doses.

This work is a nonclinical preliminary study of the effects of GNPs on melanoma cells and has been performed using gamma radiation from a cobalt source. GNPs‐loaded cancer cells have increased radiosensitivity to iodine and palladium, common radiation sources for ocular brachytherapy. Therefore, utilization of such sources in the clinical study of the effects of GNPs in the treatment of eye tumors would lead to more practical, precise, and reliable results for choroidal melanoma treatment plans.

The focus of this work was choroidal melanoma and Burkitt's lymphoma tumors. We performed *in vitro* study‎ ‎and compared the results to examine the effects of GNPs on the mentioned tumor cells. However, two unresolved issues have necessitated performing an *in vivo* study.

Firstly, given the main purpose of radiation therapy in cancer treatment, an *in vivo* study will allow us to simultaneously study the effects of GNPs on cancer cells and the nearby healthy tissues.

Secondly, delivery of NPs to target organs, or tissues, is commonly done via intravenous injection and tumors uptake the NPs via the blood circulation. Previous studies have examined the radiosensitivity of tumor cells in the presence of GNPs in prostate, skin melanoma, and liver tumors. Considering the location of the eye in the body, compared to the location of these other tumor types, it may be that a different method of GNPs delivery is required for eye tumors. An *in vivo* study can provide the optimal conditions for determining the most suitable way to deliver NPs into eye tumors.

The availability of an *in vivo* study of choroidal melanoma could lead to a better understanding of the effects of GNPs in melanoma dosimetry. A full experimental investigation of the effects of the GNPs on brachytherapy dosimetry inside the choroidal melanoma could answer many of the questions raised by this *in vitro* study. Furthermore, such analyses lead to increased reliability of results and more accurate pretreatment planning for GNPs‐based choroidal melanoma treatment.

Choroidal melanoma is structurally different from other cancer cells in which the effects of GNPs have been studied. This is the first report of the effects of GNPs on radiotherapy of eye tumors and this study was designed to physically evaluate the response of GNPs‐loaded melanoma cells to gamma irradiation.

The results showed that incubation of melanoma cells with GNP concentrations lower than 100 *μ*g/ml accompanied by gamma irradiation considerably decreased cell viability. In the absence of irradiation GNPs in such concentrations did not obviously affect the metabolism of cultured melanoma cells. The decrease in cell viability was the result of a significant increase in absorbed energy by the tumor. Moreover, it was found that concentrations of GNPs higher than 200 *μ*g/ml induced obvious cytotoxicity in melanoma cells.

Assays of GNPs cytotoxicity in Burkitt's lymphoma cells showed a slight decrease in cell viability at a concentration of 50 *μ*g/ml and clear cytotoxicity at concentrations higher than 100 *μ*g/ml.

This study examined five different concentrations of GNPs. The concentration and proper injection doses for GNPs in a sensitive tissue, such as the human eye, are important variables that need to be determined. However, further *in vitro* studies investigating various different sizes and concentrations of GNPs are required to determine the optimum conditions before any clinical or *in vivo* research can commence.

GNPs, combined with brachytherapy, are expected to be beneficial for ocular cancer patients. However, *in vivo* studies are necessary to enable precise investigation of the effects of GNPs on cancerous and healthy tissues to obtain reliable pretreatment planning for choroidal melanoma therapy. Therefore, future directions should involve a full investigation of the effects of GNPs on the choroidal melanoma dosimetry with a brachytherapy source in an animal model.

## ACKNOWLEDGMENT

All authors express their sincere thanks to the Imam Khomeini Hospital managers for their cooperation to complete this study.

## CONFLICT OF INTEREST

The authors report no conflicts of interest. The authors alone are responsible for the content and writing of the paper.

## ETHICAL APPROVAL

This article does not contain any studies with human participants or animals performed by any of the authors.
